# Upregulation of PNCK Promotes Metastasis and Angiogenesis via Activating NF-*κ*B/VEGF Pathway in Nasopharyngeal Carcinoma

**DOI:** 10.1155/2022/8541582

**Published:** 2022-04-30

**Authors:** Xiaochuan Chen, Youliang Weng, Ying Li, Wankai Fu, Zongwei Huang, Yuhui Pan, Wenquan Hong, Wanzun Lin, Xiandong Lin, Sufang Qiu

**Affiliations:** ^1^Department of Radiation Oncology, Fujian Medical University Cancer Hospital, Fujian Cancer Hospital, Fuzhou 350000, China; ^2^Department of Radiation Oncology, Shanghai Proton and Heavy Ion Center, Fudan University Cancer Hospital, Shanghai 201321, China

## Abstract

**Background:**

Distant metastasis is the major cause of treatment failure in patients with nasopharyngeal carcinoma (NPC). Thus, the identification of the molecular mechanisms and the development of novel therapeutic strategies are important. Previous studies suggest that PNCK promotes tumor growth by suppressing PI3K/AKT/mTOR signaling in NPC. However, the underlying regulatory mechanism of PNCK for NPC invasion and metastasis remains unclear.

**Methods:**

The PNCK expression level was evaluated in nonmetastatic and metastatic NPC specimens by mRNA sequencing and immunohistochemistry. In vitro migration and invasion and in vivo nude mouse metastasis model and zebrafish model were used to evaluate the effects of PNCK ectopic expression on the metastatic ability of NPC cells. Gene set enrichment and western blot analyses were used to investigate the PNCK downstream signaling pathway.

**Results:**

Human metastatic NPC samples showed elevated PNCK expression at both mRNA and protein levels. Upregulated PNCK promoted in vitro NPC cell migration, invasion, and the formation of lung metastases; the vascular-labeled fluorescence signal increased in the in vivo zebrafish model. Mechanistically, pathway analysis showed that the upregulation of PNCK may promote cell metastasis by activating the NF-*κ*B/VEGF signaling pathway.

**Conclusions:**

These findings revealed the specific critical role of PNCK in promoting NPC metastasis and angiogenesis, which suggested that PNCK may have implications as a potential therapeutic target for individualized NPC treatment.

## 1. Introduction

Nasopharyngeal carcinoma (NPC) is a common malignancy originating in the epithelium of the nasopharynx. Its incidence has a unique geographical distribution; high incidences are reported in southeast Asia and southern China [1, 2]. Due to the deep anatomical position and concealed early symptoms, 80%-90% of NPC patients are first diagnosed in the middle and advanced stages of the disease [3, 4]. Moreover, when first diagnosed, cervical lymph node metastases and aggressiveness of adjacent tissues occur in almost 75%-90% of patients owing to high invasion and early metastasis [5]. Despite the great progress in treatment strategies for NPC, including radiotherapy, which is regarded as the main treatment strategy, combined chemotherapy, targeted therapy, or immunotherapy, invasion and metastasis continue to have a key impact on the prognosis of patients [6]. The outcome for patients with metastatic NPC is very poor; the progression-free survival (PFS) period is only 6 months [7]. Thus, it is vital to further explore the molecular mechanisms underlying invasion and metastases. These findings may contribute to the development of more effective and novel options for individualized NPC therapy.

Nuclear factor-*κ*B (NF-*κ*B) is a family of transcription factors that plays a critical role in inflammation, immunity, cell proliferation, tumor-associated angiogenesis, and metastasis [7, 8] The NF-*κ*B pathway is of particular interest in cancer metastasis, as it can bind the transcription of several target genes that promote invasion and metastasis [9]. Aberrant activation of NF-*κ*B promotes cancer invasion and metastasis; it has been observed in many tumors, including breast cancer and colorectal cancer [10, 11]. However, the detailed regulatory mechanism underlying the NF-*κ*B signaling pathway in NPC metastasis remains poorly understood. Thus, the identification of new upstream regulators of NF-*κ*B signaling is of great importance for prognostic prediction and therapy.

Pregnancy upregulated nonubiquitous calmodulin (CaM) kinase (PNCK) is a unique member of the CaM kinase I family. It impacts various biological functions and plays an important role in human malignancies [12–15]. Some previous studies have focused on the role of PNCK in carcinogenesis. PNCK is indeed upregulated in breast cancer, renal carcinoma, and NPC [16–18]. PNCK is correlated with the increased proliferative properties in HER-2-positive breast cancer cells [19]. Furthermore, PNCK plays a vital role in regulating cytoplasmic and nuclear signal transduction. Thus, in recent years, diverse biological functions of PNCK have been reported [20]. In a previous study, it was found that knocking down PNCK could suppress tumor cell proliferation and promote apoptosis in NPC [16]. However, the underlying mechanism and functions of PNCK in invasion and metastases of NPC remain unclear.

The PNCK expression was evaluated in metastatic and nonmetastatic NPC tissues and it was found that PNCK expression was significantly high in the metastatic tissue. By in vitro and in vivo studies, it was found that upregulated PNCK significantly promoted the metastatic ability of NPC cells. Furthermore, it was demonstrated that PNCK facilitated the metastasis in NPC cells by the NF-*κ*B pathway and promoted NPC angiogenesis. Taken together, this study elucidates the mechanism of PNCK overexpression in promoting metastasis in NPC cells and identifies a potential prognostic biomarker and target for individualized NPC treatment.

## 2. Materials and Methods

### 2.1. Patients and Clinical Specimens

A total of 5-freshly frozen metastatic and 5-non-metastatic NPC tissue were collected by biopsy from the Fujian Cancer Hospital between January 2015 and March 2015. Gene expression analysis was performed using 132 paraffin-embedded NPC specimens obtained from patients diagnosed with primary NPC and who did not undergo any treatments before the biopsy (Supplemental Files 1). Two senior pathologists who were blind to patients' clinical information confirmed the pathology. Protocols for sample collection were performed with the approval of the ethics committee of Fujian Medical University Cancer Hospital, Fujian Cancer Hospital. All patients had signed the written informed consent.

In an independent NPC dataset (GSE102349 from GPL11154 platform) from the Gene Expression Omnibus (GEO) (https://www.ncbi.nlm.nih.gov/gds/), 113 patient data were included as per the following selection criteria: (a) definite histologically diagnosis of NPC; (b) availability of RNA-seq data (Supplemental Files 2).

### 2.2. Cell Culture and Transfection

Two human NPC cell lines (CNE-2 and SUNE-1) were obtained from the Shanghai Institutes for Biological Sciences. All cells were cultured in RPMI 1640 medium supplemented with 10% fetal bovine serum (FBS), 100 U/mL penicillin, and 100 U/mL streptomycin and maintained at 37°C in a 5% CO_2_ humidified incubator. To generate PNCK overexpression and stable knockdown transfectants, lentiviral vectors expressing GFP or control vector plasmids (pcDNA3.1-cofilin) in CNE-2 and SUNE-1 cells were introduced (Supplementary Figure 1). The plasmid and packaged lentivirus constructs were commercially synthesized (Genechem Co. Ltd., Shanghai, China).

### 2.3. RNA-seq Data Analysis

The freshly frozen tissues and CNE-2 cells were used for mRNA sequencing. Total RNA was extracted with TRIzol (Invitrogen), and its quality was assessed with Agilent 2100 BioAnalyzer (Agilent Technologies, Santa Clara, CA, USA) and Qubit Fluorometer (Invitrogen). hg38 was used as the reference assembly for the human genome. The sequencing quality was assessed using the quality assessment software, FastQC (v0.11.5); the low-quality reads were removed using NGSQC (v2.3.3) [21, 22]. The clean paired-end reads were then mapped onto the reference genome using HISAT2 (v2.1.0) with default parameters [23]. Gene expression levels were calculated using StringTie (v1.3.3b) [24]. The differentially expressed genes (DEGs) between the samples were evaluated by DESeq (v1.28.0) [25]. Thousands of independent statistical hypothesis testing were conducted separately for the DEGs. FDR-corrected *P* value (FDR *P*) < 0.05 was used as a cutoff for statistical significance. Parameters for defining significant DEGs were ≥2-fold change (|log2 *FC*| ≥ 1 (FC, the fold change of expressions)) in the transcript data, and *P* ≤ 0.05. The DEGs were annotated based on the information acquired from the ENSEMBL, NCBI, UniProt, GO, and KEGG databases. The biological processes associated with the DEGs were identified using the Ingenuity Pathway Analysis (IPA) Software (QIAGEN Inc., https://www.qiagenbioinformatics.com/products/ingenuity-pathway-analysis). Volcano plots were generated with the “pheatmap” and “ggplot2” packages in R.

### 2.4. Immunohistochemistry (IHC) Analysis

The paraffin-embedded NPC tissue sections were analyzed by IHC using the anti-human PNCK antibody (Abcam, Cat #ab235093). Following the DAB treatment, HRP-linked secondary antibodies (Abcam, Cat #ab205718, UK) were used. Stained cells were blindly scored based on the staining intensity (negative: 0; weak: 1; moderate: 2; and strong: 3), and the abundance of positive cells (0%: 0; 0-5%: 1; 6-25%: 2; 26-50%: 3; 51-75%: 4; and ≥76%: 5) was validated by two senior pathologists. To obtain the final score, the intensity score (0-3) was multiplied by the extent score (0-5) to determine the levels of PNCK expression.

### 2.5. Real-Time PCR and Western Blotting

PCR and WB were performed as previously described [26]. We used the following PCR primers: forward PNCK-F: 5′-TGACATCTCAGAATCAGCCAAAG-3′, reverse PNCK-R 5′-GTGTCCGAGCAAAGTTCTTCC-3′; VEGFA-F: 5′-GGAGGGCAGAATCATCACGA-3′, reverse VEGFA-R 5′-GCTCATCTCTCCTATGTGCTGG-3′; the following primary antibodies were used: NF-*κ*B P65 (1 : 1000, CST, USA), NF-*κ*B p-P65 Ser536 (1 : 1000, CST, USA), p-I*κ*B Ser32 (1 : 1000, CST, USA), PNCK (1 : 500, Sigma, USA), VEGFA (1 : 1000, Proteintech, China), Histone-H3 (1 : 10000, CST, USA), and GAPDH (1 : 10000, Santa Cruz, USA).

### 2.6. Gene Set Enrichment Analysis

GSEA was used to compare the levels of expression enrichment between an a priori defined set of genes and the high- and low-PNCK expression groups using the MSigDB Collection (c2.cp.kegg and c5.go.bp. v7.2. symbols.gmt). High- and low-PNCK were used as the phenotype label, and the gene set permutations were performed 1000 times for each analysis. False discovery rate (FDR) and normalized enrichment score (NES) were used to classify the GO and KEGG pathways enriched in differential phenotypes.

### 2.7. Wound Healing Assay

Transfected CNE-2 and SUNE-1 cells (~1 × 10^5^ cells/well) were seeded into six-well plates and cultured till they attained 80% confluence. Then, the monolayers were wounded by dragging a 1 mL pipette tip across the cells. The cells were then washed to remove debris and were allowed to migrate for 48 h. Following wounding, cells were imaged at 0 h and 48 h using a DMI 6000 inverted microscope. Cell migration was quantified using the ImageJ software as the wound healing index, i.e., the wound area healed by the cells at 48 h after scratching relative to the wound area at 0 h.

### 2.8. Transwell Assay

Transfected cells were digested and resuspended in serum-free RPMI 1640. Then, 200 *μ*L of the cell suspension was seeded (1 × 10^6^ cells/mL) into the upper chambers of an 8 *μ*m transwell filter (Millipore), precoated with 1 : 3 diluted Matrigel (BD Biosciences). RPMI 1640 supplemented with 10% FBS (700 *μ*L) was added to the lower chamber. Subsequently, the plate was incubated for 24 h at 37°C and 5% CO_2_. The migration and invasive ability of the cells were assessed based on the penetration of the membrane and gel membrane of the matrix, respectively. Following 24 h incubation, the residual cells in the upper chamber were removed with cotton swabs and dried. Then, migrated and invaded cells were fixed with 4% paraformaldehyde for 15 min at room temperature, washed with PBS, and stained with 0.1% crystal violet for 10 min at room temperature. Cells were counted in at least 5 randomly selected fields of view observed under a DMI 6000 inverted microscope (×200).

### 2.9. In Vivo Experimental Metastasis Assay

Male BALB/c (nu/nu) nude mice (4-6 weeks of age) were purchased from Wushi Experimental Animal Supply Co., Ltd. (Fuzhou, China). Metastasis of PNCK-overexpressing CNE-2 cells and wild-type cells was injected intravenously in the tail vein. The experiment was terminated 28 days after tumor cell implantation. Then, the lungs were embedded in paraffin and H&E staining was performed after euthanizing the animals. Metastatic nodules in the lungs of mice were counted.

### 2.10. Zebrafish Model and Microinjection

For PNCK overexpression, the one-cell-stage embryos of Tg (fli1a: EGFP) transgenic line with vascular fluorescent labeling, were microinjected with CMV-derived human PNCK gene overexpression vector CMV-hPNCK (about 200-300 pg/embryo) using a microinjection apparatus (Eppendorf, FemtoJet 4i). For knockdown of PNCK, RNA-targeted editing technology through the CRISPR-RfxCas13d (CasRx) system was used [27]. Briefly, the endogenous PNCK gene of zebrafish was knocked down by coinjection with 2-3 nl CasRx capped mRNA (200 ng/*μ*L) and gRNA (100 ng/*μ*L). After 48 hours of injection, the overall fluorescence signal was measured using a stereoscopic fluorescence microscope (SMZ800N). The ImageJ software was used to evaluate the formation of vascular structure and length of intersegmental vessels (ISVs). Moreover, the antiangiogenic or angiogenesis activity was identified based on the total inhibition of ISV growth and incomplete sprouting of the ISVs from the dorsal aorta (DA) to the dorsal longitudinal anastomotic vessel (DLAV).

### 2.11. Statistical Analysis

SPSS 17.0 and R software (Version 3.4.4) were used for all statistical analyses. The quantitative data from at least three independent experiments are presented as mean ± SD. Student's *t*-test and ANOVA were used to compare the differences between the groups. Spearman's rho test was used to analyze the correlation among the qualitative parameters. All differences in data were determined as statistically significant at *P* < 0.05.

## 3. Results and Discussion

### 3.1. PNCK Is Upregulated in Metastatic Nasopharyngeal Carcinoma Tissue

**Figure 1:**
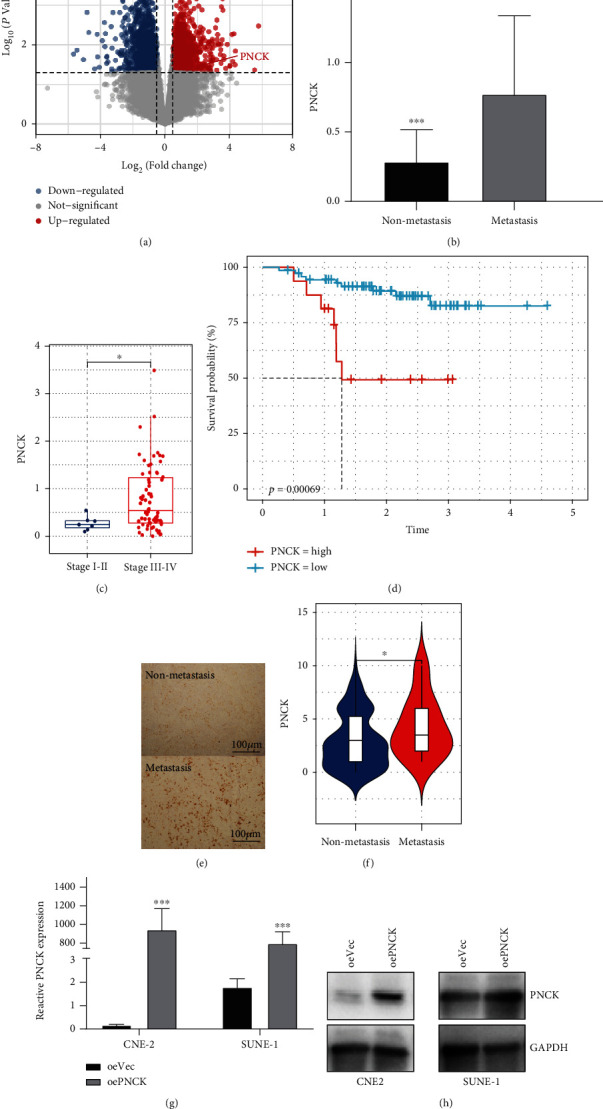
High expression of PNCK in NPC metastatic tissue. (a) The volcanic plot shows the distribution of significantly upregulated or downregulated differentially expressed genes using 5-metastatic and 5-non-metastatic NPC tissue samples; PNCK is among the upregulated genes. (b) Relative expression of PNCK evaluated by mRNA sequencing. (c) GSE102349 cohort indicating the higher PNCK expression in advanced stage (stage III-IV) as compared to the early-stage NPC patients (stage I-II). (d) GSE102349 cohort indicating that the higher PNCK expression is associated with worse PFS. (e) Validation of the PNCK protein expression using paraffin-embedded NPC nonmetastatic and metastatic tissues assessed by immunohistochemistry, and (f) the quantification of PNCK. Student's *t*-test, ^∗^*P* < 0.05. (g, h) The expression of PNCK levels in NPC cells was evaluated with PNCK overexpression (oePNCK) by PCR and WB.

To investigate the role of PNCK in NPC metastasis, mRNA expression profiles were analyzed by mRNA sequencing of 5-metastatic and 5-non-metastatic NPC tissues. It was found that 520 and 808 mRNAs were upregulated and downregulated, respectively, according to the criteria of *P* < 0.05 and |log2 *FC*| > 1.0 threshold values (Figure 1(a)). It was found that the mean level of PNCK expressed in metastatic samples was twice as high as in the nonmetastatic control tissues (Figure 1(b)). The independent cohort, GEO dataset, GSE102349, was used to validate the expression levels of PNCK, and it was found that it was significantly higher in the advanced stage (III-IV) as compared to NPC patients in the early stage (I-II) (*P* < 0.05, Figure 1(c)). Moreover, higher expression of PNCK was significantly associated with worse PFS which indicated poor prognosis (Figure 1(d)). Additionally, the protein level expression of PNCK was further evaluated for its clinical significance in NPC patients using immunohistochemical staining of 132 paraffin-embedded NPC tissues (Figure 1(e)). Overall, higher PNCK protein levels in metastatic as compared to nonmetastatic samples were found (*P* < 0.05, Figure 1(f)). Furthermore, there were no significant associations of PNCK expression with age, sex, T stage, N stage, or TNM stage (all *P* > 0.05). However, PNCK levels were significantly correlated with distant metastasis (*P* = 0.047, Table 1).

### 3.2. PNCK Promotes In Vitro and In Vivo Cell Metastasis in NPC

**Figure 2:**
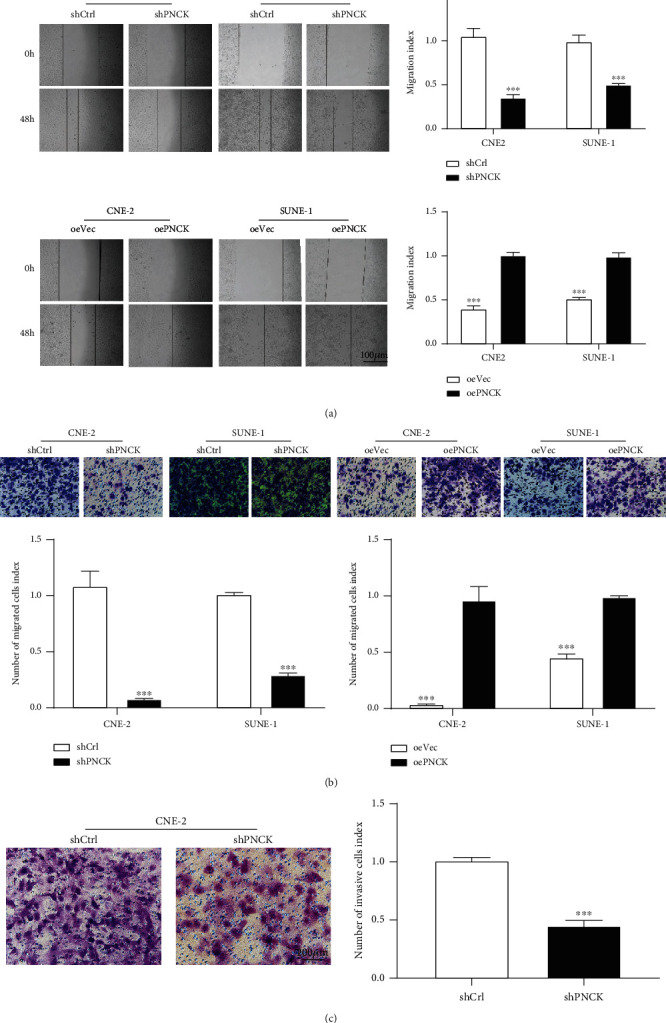
Representative results and quantification of in vitro experiments in generated NPC cell lines with PNCK overexpression (oePNCK) and PNCK knockdown (shPNCK). (a) PNCK knockdown inhibits NPC cell migration distance, while overexpression of PNCK promotes cell migration as seen in wound healing assays. (b, c) PNCK overexpression enhances the invasion and migration potential of NPC cells as assessed by transwell assays. Student's *t*-test, ^∗∗^*P* < 0.01 and ^∗∗∗^*P* < 0.001.

Stably overexpressing PNCK-NPC cell lines (oePNCK cells) were generated using CNE-2 and SUNE-1 cells to assess the PNCK-mediated metastatic abilities in NPC. The mRNA and protein levels were confirmed by real-time PCR and western blot analysis (Figures 1(g) and 1(h)). It was shown that PNCK silencing inhibited NPC cell migration and invasion. In contrast, PNCK overexpression enhanced the metastatic potential of cells as observed in the wound-healing and transwell invasion assays (Figures 2(a)–2(c), *P* < 0.01).

**Figure 3:**
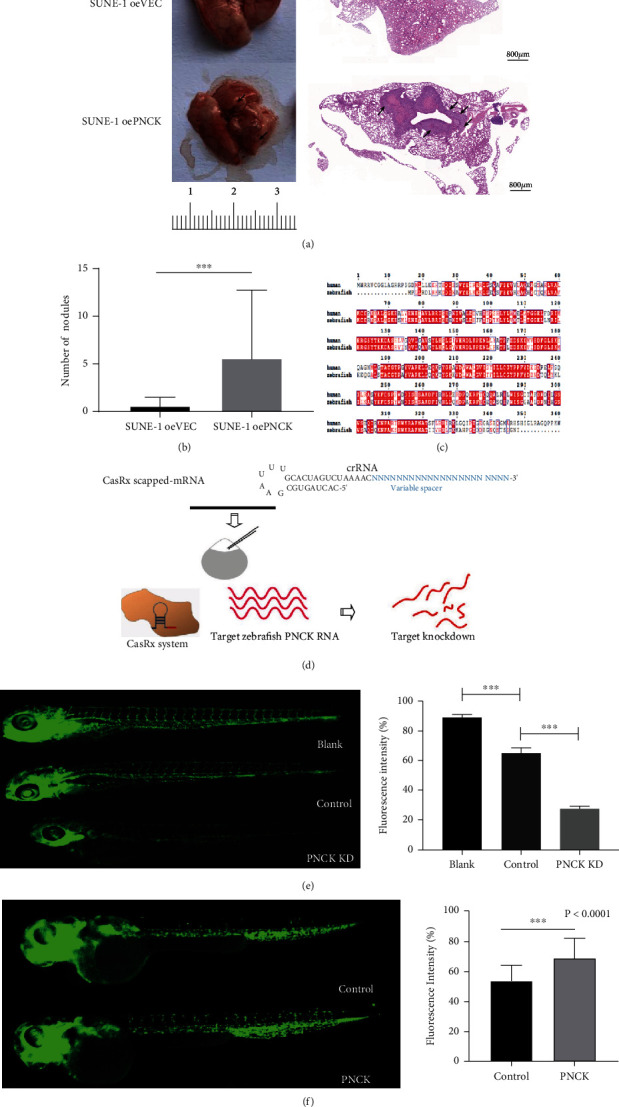
Role of PNCK in the in vivo metastasis and angiogenesis model of NPC. (a) Representative images and (b) quantification of lung metastatic foci in mice after injecting SUNE-1 cells with overexpression of PNCK or control vector by tail vein injection. Arrows indicate surface metastatic nodules. (c) Alignment of PNCK amino acid sequences of human and zebrafish. (d) The schematic diagram of RNA-editing technology in the zebrafish embryo. (e) Fluorescence intensity of intersegmental vessels in Tg (fli1a: EGFP) embryos inoculated with blank, control, or PNCK KD vector. ANOVA, ^∗∗∗^*P* < 0.001. (f) Fluorescence intensity of intersegmental vessels in Tg (fli1a: EGFP) embryos inoculated with control or PNCK vector. Student's *t*-test, ^∗∗∗^*P* < 0.001.

The above results were validated in an in vivo experiment with a nude mouse model for lung metastasis. SUNE-1 cells transfected with control or PNCK overexpression vector were inoculated into nude mice (6 weeks old) by tail vein injection. Then, the number of metastatic lesions in the lungs after 4 weeks was evaluated. The metastatic foci on the lung surface were significantly higher in the oePNCK group than those in the oeVec group, as shown in Figures 3(a) and 3(b) (*P* < 0.001). Similarly, the numbers and volumes of micrometastases in the lungs of mice injected with SUNE-1 cells with overexpressing PNCK were significantly high (Figure 3(a)). These results suggested that PNCK is important for promoting metastases in NPC cells.

### 3.3. PNCK Is Required for Angiogenesis in Zebrafish

Zebrafish is a powerful model system that is increasingly being used for cancer research, drug discovery, and xenografting of cancer cells [28]. Recent studies suggest the possibility of using the zebrafish model to investigate tumor angiogenesis [29]. Through bioinformatic analysis, it was found that the PNCK gene of zebrafish has more than 60% homology with the human gene (Figure 3(c)). As the VEGF signaling pathway was significantly enriched in the PNCK-high expression group, RNA-targeted editing technology was used to knock down the endogenous PNCK gene using Tg (fli1a: EGFP) transgenic zebrafish embryos to further verify the potential correlation between PNCK expression and angiogenesis (Figure 3(d)). When the treated embryos developed to 48 hours postfertilization (hpf) stage, the overall fluorescence signal of zebrafish was measured by a fluorescence microscope. Through calculation and statistical analysis, it was found that angiogenesis in intersegmental vessels (ISVs) of zebrafish was downregulated after the knockdown of PNCK gene as compared to the control group (Figure 3(e)). Similarly, to inhibit angiogenesis, the PNCK overexpression plasmid (CMV-hPNCK) and control (CMV-mCherry) were injected into Tg (fli1a: EGFP) transgenic zebrafish embryos, which were treated with 0.75 *μ*mol/L sunitinib; embryos with a high level of PNCK had upregulated angiogenesis in ISVs at 48 hpf (Figure 3(f)).

### 3.4. NF-*κ*B/VEGF Signaling Pathway Activation Is Upregulated by PNCK Overexpression in NPC Cells

**Figure 4:**
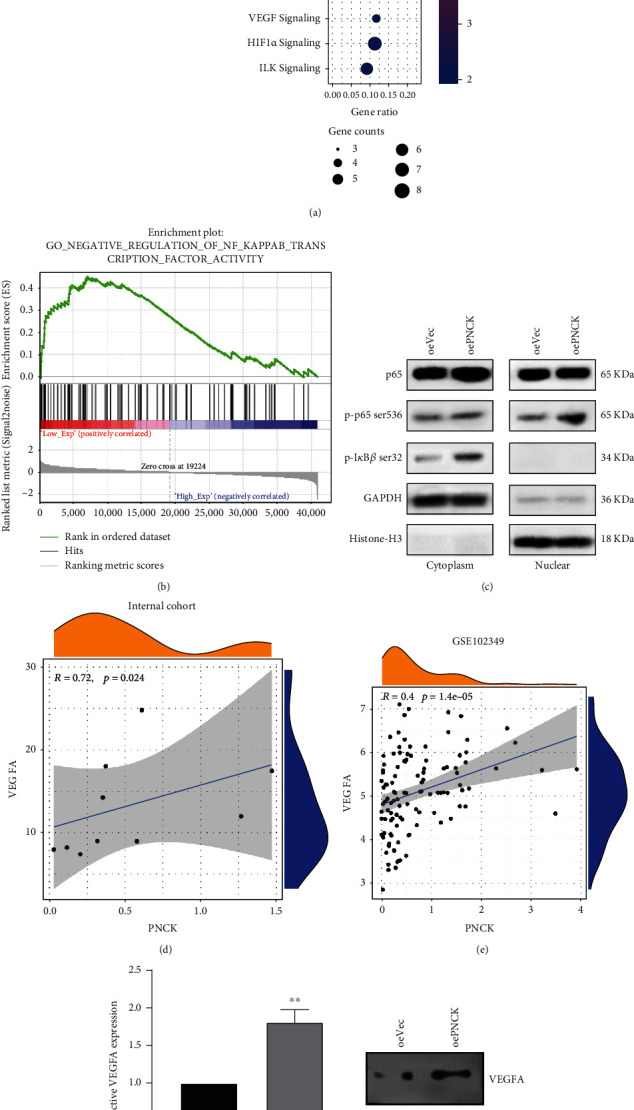
The activity of the NF-*κ*B/VEGF signaling pathway is upregulated by PNCK overexpression in NPC cells. (a) Dot plot of Ingenuity Pathway Analysis (IPA) shows significant changes in NF-*κ*B and VEGF signaling pathways. (b) GSEA shows negative regulation of NF-*κ*B transcription factor activity enriched in the PNCK-low expression phenotype. (c) WB was used to investigate the P65, p-P65, p-I*κ*B, GAPDH, and Histone-H3. The correlation between PNCK and VEGFA in (d) internal cohort and (e) GSE102349 cohort. (f, g) The expression of VEGFA levels in NPC cells was evaluated by PCR and WB.

To elucidate the downstream signaling pathway of PNCK regulation of metastasis phenotype in NPC, a genome-wide expression analysis was performed to determine the DEGs in PNCK knockdown-CNE-2 cell lines [16]. Pathway analysis using Ingenuity Pathway Analysis (IPA) showed that NF-*κ*B and VEGF signaling pathways were significantly altered (Figure 4(a)). Likewise, GSEA was performed to identify the GO annotations and enriched signaling pathways between low- and high-PNCK expression groups using 5-metastatic and 5-non-metastatic NPC tissues by microarray. As shown in Figure 4(b), negative regulation of NF-*κ*B transcription factor activity was higher in the PNCK-low expression phenotype. Further investigation revealed that once the cells were treated with a P65 activator, tumor necrosis factor *α* (TNF-*α*), at 10 ng/mL for 30 min, P65 was rapidly phosphorylated in both nuclei and cytoplasm of PNCK-overexpressed CNE-2 cells. Furthermore, phosphorylated I*κ*B was accumulated in PNCK-overexpressed cells after TNF-*α* stimulation (Figure 4(c)), suggesting that the NF*κ*B pathway was activated by PNCK.

Angiogenesis, the recruitment of new blood vessels, is essential in the metastatic pathway [30]. Tumor angiogenesis is regulated by the production of angiogenic stimulators including the members of the vascular endothelial growth factor families (VEGF) [31]. Previous studies show that an increase in expression of NF-*κ*B contributes to tumor angiogenesis in colorectal cancer. VEGF may play an important role in mediating the NF-*κ*B angiogenic pathway [32]. Although the VEGF pathway was not significantly enriched in the high-PNCK group, the expression of PNCK was positively associated with VEGFA as shown in Figure 4(d) (*r* = 0.72, *P* = 0.024). VEGFA is a key facilitator of angiogenesis in cancer [26]. GEO datasets verified the significant relationship between PNCK and VEGFA (*r* = 0.4, *P* < 0.001, Figure 4(e)). PCR and WB results also showed that the expression of VEGFA in CNE-2 cells increased with PNCK overexpression as compared to the negative control (Figures 4(f) and 4(g)). Collectively, these findings suggested that downregulation of PNCK may inhibit the NF-*κ*B/VEGF signaling pathway in NPC.

## 4. Discussion

NPC is a malignancy with a high potential of invasion and metastasis; distant metastasis is a major cause of treatment failure [33, 34]. Hence, a better understanding of the molecular mechanism of tumor metastasis is crucial to develop effective treatment strategies for NPC patients. In this study, based on mRNA sequencing and IHC results, it was confirmed that PNCK is highly expressed in metastatic NPC specimens. By analyzing the GEO cohort, it was further validated that PNCK was highly expressed in advanced NPC patients and was associated with PFS in NPC. The upregulation of PNCK promoted in vitro migration and invasion of NPC cells and led to the development of lung metastasis in vivo. Additionally, experiments in zebrafish showed that PNCK overexpression promoted tumor angiogenesis. The results also highlighted the important role of the NF-*κ*B/VEGF signaling pathway in NPC metastasis.

PNCK is located on chromosome Xq28. It is a member of the CaM kinase family and has the enzymatic activity of serine/threonine kinases [15]. It is involved in various biological functions including transcriptional regulation, cell-cycle control, and neurotransmitter release [13, 14]. Some previous studies focus on the role of PNCK in cancers [16, 18, 35]. For example, in hepatocellular carcinoma, PNCK was highly expressed in the tumor as compared to the nontumor samples at both mRNA and protein levels; it is related to poorer prognosis, higher Edmondson grade, higher AFP levels, microvascular invasion, and intrahepatic metastasis [35]. Additionally, high PNCK expression is associated with poor prognosis, advanced T, N stage, and poor differentiation in clear cell renal cell carcinoma [18]. Previously, it was demonstrated that NPC tumor tissues had elevated PNCK expression, and it could regulate cell proliferation and apoptosis through the PI3K/AKT/mTOR signaling pathway [21]. However, the underlying mechanisms and clinical value of PNCK in tumor metastasis have not been elucidated.

Invasion and metastasis are the major contributors to treatment failures for most cancers [36]. Distant metastasis further limits the improvements in NPC treatment [34]. GSEA and IPA analysis were used to elucidate the potential role of PNCK in NPC metastasis by comparison with PNCK-depleted cells. It was found that the PNCK-high expression phenotype had enriched the NF-*κ*B/VEGF signaling pathway. NF-*κ*B is critical for the regulation of tumor cell metastasis [9]. Liang et al. and Zhang et al. show that CAPE and NKILA repress NPC metastasis through NF-*κ*B pathway inhibition [37, 38]. However, these studies did not address the issue of angiogenesis. Angiogenesis is a key process in cancer spread and metastasis [39]. Oxygen and other nutrients from vasculature are required for metastases [40]. Previous studies indicate that the increased cytosolic Ca^2+^ activates PNCK, which in turn phosphorylates I*κ*B*α* at Ser32 and triggers Ca^2+^-dependent NF-*κ*B signaling activity under hypoxic conditions [41]. The activated Ca^2+^-induced NF-*κ*B pathway promotes tumor progression by increasing the transcription of VEGF and thus promoting angiogenesis and cancer development [41]. In this study, we have observed that the NF-*κ*B pathway was significantly enriched in the high PNCK group. Upregulation of PNCK may induce the phosphorylation of I*κ*B*α* at Ser32, which further induced I*κ*B*α* degradation and NF-*κ*B activation in NPC. The underlying mechanisms of these roles need to be further studied. Moreover, the zebrafish model was used to validate the potential role of PNCK-mediated angiogenesis in NPC in the present study. Interestingly, it was found that increased PNCK expression promoted the outgrowth of ISVs of zebrafish, which indicated positive regulation of NPC angiogenesis.

Nevertheless, there are certain limitations of the current study. First, the sample size of the sequencing cohort was small. However, at the protein level, a larger tissue sample size was included. Second, due to the lack of access to the complete clinical data, the evaluation was limited to the association with PNCK expression and progression-free survival only. Third, it was found that NF-*κ*B/VEGF signaling pathways play a critical role in NPC metastasis, but the detailed mechanism of regulation by PNCK and the corresponding upstream and downstream targets remain unclear. Therefore, further studies are needed to elucidate the mechanisms underlying the interactions between PNCK and the NF-*κ*B/VEGF axis in NPC metastasis.

## 5. Conclusion

In conclusion, it was confirmed that PNCK expression was high in metastatic tissues and was associated with PFS in NPC. PNCK promoted NPC cell migration, invasion, and angiogenesis by the activation of the NF-*κ*B/VEGF signaling pathway. These data provide new insights for PNCK as a useful indicator to predict future metastasis and as an efficient antiangiogenic target for NPC treatment. Further studies are needed to better understand the molecular mechanisms underlying the interactions between PNCK and NF-*κ*B/VEGF axis in NPC metastasis.

## Figures and Tables

**Table 1:** Clinical characteristics of NPC patients according to high and low PNCK expression.

Characteristics	No. of patients	Expression of PNCK		*P* value
		Low, *n* (%)	High, *n* (%)	
Age				
≤45	63	46 (47.9)	17 (47.2)	>0.999
>45	69	50 (52.1)	19 (52.8)	
Sex				
Male	103	75 (78.1)	28 (77.8)	>0.999
Female	29	21 (21.9)	8 (22.2)	
T stage				
T1-T2	51	39 (40.6)	12 (33.3)	0.548
T3-T4	81	57 (59.4)	24 (66.7)	
N stage				
N0-N1	41	28 (29.2)	13 (36.1)	0.527
N2-N3	91	68 (70.8)	23 (63.9)	
TNM stage				
I-II	10	8 (8.3)	2 (5.6)	0.589
III	86	64 (66.7)	22 (61.1)	
IV	36	24 (25.0)	12 (33.3)	
Distant metastasis				
No	112	83 (86.5)	29 (80.6)	0.047
Yes	20	13 (13.5)	7 (19.4)	

## Data Availability

The public dataset used in this study is freely available at https://www.ncbi.nlm.nih.gov/geo/.

## Abbreviations

NPC: Nasopharyngeal carcinoma
PNCK: Pregnancy-upregulated nonubiquitous calmodulin kinase
VEGF: Vascular endothelial growth factor
HER-2: Human epidermal growth factor receptor-2
GEO: Gene Expression Omnibus
GO: Gene Ontology
KEGG: Kyoto Encyclopedia of Genes and Genomes
ISV: Intersegmental vessel
DLAV: Dorsal longitudinal anastomotic vessel
PFS: Progression-free survival.

## Supplementary Materials

Supplementary Materials The levels of PNCK expression from 132 paraffin-embedded NPC specimens are presented in Supplemental Files 1. Supplemental Files 2 shows the patients' PNCK expression and corresponding clinical follow-up information from the NPC dataset GSE102349. Supplementary Figure 1 shows the schema of the plasmids constituting the lentiviral vector.
