# Supplementary figures and images for: Upregulation of PNCK Promotes Metastasis and Angiogenesis via Activating NF-κB/VEGF Pathway in Nasopharyngeal Carcinoma
Source: J Oncol. 2022 Apr 30;2022:8541582. doi: 10.1155/2022/8541582 (PMC9078829; doi:10.1155/2022/8541582)

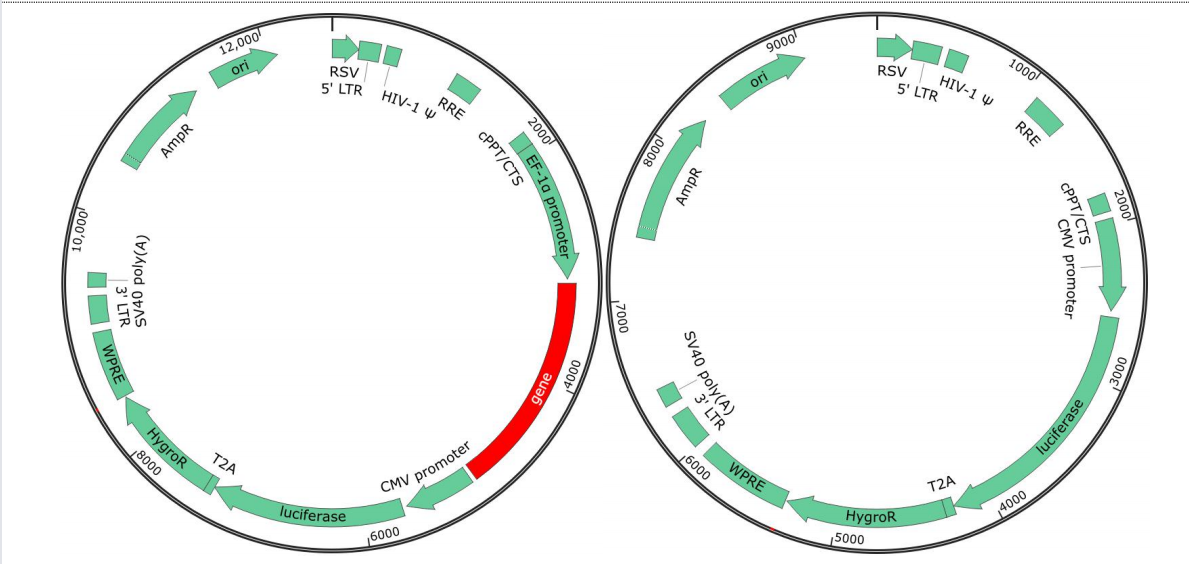


Supplementary figure 1 The plasmid (pcDNA3.1-cofilin) of PNCK overexpression.

Supplement: Supplementary Materials — The levels of PNCK expression from 132 paraffin-embedded NPC specimens are presented in Supplemental Files 1. Supplemental Files 2 shows the patients' PNCK expression and corresponding clinical follow-up information from the NPC dataset GSE102349. Supplementary Figure 1 shows the schema of the plasmids constituting the lentiviral vector. [file 8541582.f1.docx]
